# Supplementary material for: Phosphoproteomics of ATR signaling in mouse testes
Source: eLife. 2022 Feb 8;11:e68648. doi: 10.7554/eLife.68648 (PMC8824463; doi:10.7554/eLife.68648)
Supplement: Transparent reporting form [file elife-68648-transrepform1.pdf]

eLIFE
1st Floor
24 Hills Road
Cambridge CB2 1JP, UK
P 01223 855340
W elifesciences.org
T @elife

eLife's transparent reporting form

We encourage authors to provide detailed information *within their submission* to facilitate the interpretation and replication of experiments. Authors can upload supporting documentation to indicate the use of appropriate reporting guidelines for health-related research (see EQUATOR Network), life science research (see the BioSharing Information Resource), or the ARRIVE guidelines for reporting work involving animal research. Where applicable, authors should refer to any relevant reporting standards documents in this form.

If you have any questions, please consult our Journal Policies and/or contact us: editorial@elifesciences.org.

Sample-size estimation

- You should state whether an appropriate sample size was computed when the study was being designed
- You should state the statistical method of sample size computation and any required assumptions
- If no explicit power analysis was used, you should describe how you decided what sample (replicate) size (number) to use

Please outline where this information can be found within the submission (e.g., sections or figure legends), or explain why this information doesn't apply to your submission:

Since each phosphoproteomic analysis of each biological replicate employed in this work required extensive access to a mass spectrometer (which is cost limited), we limited the number of biological replicates for each comparison and prioritized the 4 hours ATRi treatment experiment. We used 5 replicates for the 4 hour ATRi treatment, 2 replicates for the 2.5-3 day ATRi treatment, and 3 replicates for the RAD1-cKO experiment, in which each biological replicate corresponds to testes from different mice.

Replicates

- You should report how often each experiment was performed
- You should include a definition of biological versus technical replication
- The data obtained should be provided and sufficient information should be provided to indicate the number of independent biological and/or technical replicates
- If you encountered any outliers, you should describe how these were handled
- Criteria for exclusion/inclusion of data should be clearly stated
- High-throughput sequence data should be uploaded before submission, with a private link for reviewers provided (these are available from both GEO and ArrayExpress)

Please outline where this information can be found within the submission (e.g., sections or figure legends), or explain why this information doesn't apply to your submission:

eLife Sciences Publications, Ltd is a limited liability non-profit non-stock corporation incorporated in the State of Delaware, USA, with company number 5030732, and is registered in the UK with company number FC030576 and branch number BR015634 at the address 1st Floor, 24 Hills Road, Cambridge CB2 1JP | August 2014

For the proteomic datasets, information on replicates is found within the text in the results and methods sections. For meiotic spread immunohistochemistry, information in replicates and sample size (number of cells quantified) is found in the figure legends with data from individual biological replicates explicitly shown in supplemental figures S1A, S3C, S3B, S7A, S7C, S8A, S8C, and S10A. To illustrate the variation in the immunohistochemistry experiments on meiotic spreads, additional examples of quantified images can be found in figures S1B, S3C, S7B, S7D, S8B, S8D and S10B. Further clarification can be found in the methods section. In order to comply with ARRIVE guidelines for reporting work involving animals, mouse strain background and age are also referenced in the methods section and/or corresponding figure legend. As stated in the methods section, mouse handling for these experiments was done following federal and institutional guidelines that underwent approval by the Institutional Animal Care and Use Committee (IACUC) at Cornell University.

Statistical reporting

- Statistical analysis methods should be described and justified
- Raw data should be presented in figures whenever informative to do so (typically when N per group is less than 10)
- For each experiment, you should identify the statistical tests used, exact values of N, definitions of center, methods of multiple test correction, and dispersion and precision measures (e.g., mean, median, SD, SEM, confidence intervals; and, for the major substantive results, a measure of effect size (e.g., Pearson's r, Cohen's d)
- Report exact p-values wherever possible alongside the summary statistics and 95% confidence intervals. These should be reported for all key questions and not only when the p-value is less than 0.05.

Please outline where this information can be found within the submission (e.g., sections or figure legends), or explain why this information doesn't apply to your submission:

Information on statistical analysis for all meiotic spread immunohistochemistry quantifications can be found in the figure legends. Details about how proteomic data was analyzed, organized and prioritized can be found in the methods sections.

(For large datasets, or papers with a very large number of statistical tests, you may upload a single table file with tests, Ns, etc., with reference to sections in the manuscript.)

Group allocation

- Indicate how samples were allocated into experimental groups (in the case of clinical studies, please specify allocation to treatment method); if randomization was used, please also state if restricted randomization was applied
- Indicate if masking was used during group allocation, data collection and/or data analysis

Please outline where this information can be found within the submission (e.g., sections or figure legends), or explain why this information doesn't apply to your submission:

eLife Sciences Publications, Ltd is a limited liability non-profit non-stock corporation incorporated in the State of Delaware, USA, with company number 5030732, and is registered in the UK with company number FC030576 and branch number BR015634 at the address 1st Floor, 24 Hills Road, Cambridge CB2 1JP | August 2014

For mice treated with ATR inhibitors, we monitored the disappearance of gammaH2AX at the sex body by immunofluorescence in meiotic spreads as an indication that the treatment regime was effective in inhibiting ATR in spermatocytes. Mice with impaired gammaH2AX staining were allocated into the ATR inhibitor experimental group, and distinguished from control, untreated mice.

Additional data files ("source data")

- We encourage you to upload relevant additional data files, such as numerical data that are represented as a graph in a figure, or as a summary table
- Where provided, these should be in the most useful format, and they can be uploaded as "Source data" files linked to a main figure or table
- Include model definition files including the full list of parameters used
- Include code used for data analysis (e.g., R, MatLab)
- Avoid stating that data files are "available upon request"

Please indicate the figures or tables for which source data files have been provided:

The raw mass spectrometry phosphoproteomic data generated in this study were deposited to the Massive database (http://massive.ucsd.edu) and received the ID: MSV000086764, doi:10.25345/C57N54, and ProteomeExchange ID: PXD023803.

An in-house R-script was used for calculation of prevalence of amino acids surrounding identified phosphorylation sites, and explained in the legend of Figure 9A-B. Code used for data analysis in this manuscript can be found at https://github.com/gerardoarroyomartinez/911_ATR-Phosphoproteomics.git
